# Humanistic care relieves mental distress of inpatients in the shelter hospital during COVID-19 pandemic in Shanghai: a cross-sectional observational study

**DOI:** 10.3389/fpsyt.2023.1178834

**Published:** 2023-07-27

**Authors:** Dongdong Xiao, Hua Huang, Min Chen, Jieying Wang, Wei Zhai, Jiaqi Ye, Minjie Chen, Weilin Fang, Yeqian Zhang, Zhiwei Fu, Yifei Shen, Ziji Yan, Chenlu Shen, Jun Qin, Yanli Luo, Junhua Zheng

**Affiliations:** ^1^Department of Urology, Ren Ji Hospital, School of Medicine, Shanghai Jiao Tong University, Shanghai, China; ^2^Department of Administration, Ren Ji Hospital, School of Medicine, Shanghai Jiao Tong University, Shanghai, China; ^3^Department of Nursing, Ren Ji Hospital, School of Medicine, Shanghai Jiao Tong University, Shanghai, China; ^4^Clinical Center for Investigation, Ren Ji Hospital, School of Medicine, Shanghai Jiao Tong University, Shanghai, China; ^5^Department of Outpatient and Emergency Management, Ren Ji Hospital, School of Medicine, Shanghai Jiao Tong University, Shanghai, China; ^6^Department of Gastrointestinal Surgery, Ren Ji Hospital, School of Medicine, Shanghai Jiao Tong University, Shanghai, China; ^7^Department of Orthopaedics, Ren Ji Hospital, School of Medicine, Shanghai Jiao Tong University, Shanghai, China; ^8^Trade Union, Ren Ji Hospital, School of Medicine, Shanghai Jiao Tong University, Shanghai, China; ^9^Department of Logistics, Ren Ji Hospital, School of Medicine, Shanghai Jiao Tong University, Shanghai, China; ^10^Department of Psychological Medicine, Ren Ji Hospital, School of Medicine, Shanghai Jiao Tong University, Shanghai, China

**Keywords:** COVID-19, mental distress, shelter hospital, inpatients, humanistic care

## Abstract

**Objective:**

The prevalence of mental distress has been noted in shelter hospitals set up for COVID-19. Potential risk demographic and hospitalization factors were screened. We also aimed to determine whether humanistic care established in the shelter hospital was effective in ameliorating mental distress.

**Methods:**

A cross-sectional observational survey-based single-centered study was conducted from 28th April to 5th May 2022 during the COVID-19 pandemic in Shanghai. Asymptomatic adult inpatients and those with mild symptoms were recruited for this study, and humanistic care measures were carried out by the administrative office according to the Work Program on Psychological Assistance and Social Work Services at the Shelter Hospital launched on 5th March 2020. Symptoms of mental distress, such as reported stress, anxiety, depression, and insomnia were measured using the Chinese Stress Response Questionnaire-28, the Chinese version of Generalized Anxiety Disorder-7, Patient Health Questionnaire-9, and Insomnia Severity Index-7, respectively.

**Results:**

In total, 1,246 out of 9,519 inpatients, including 565 (45.35%) women and 681 (54.65%) men, with a median age of 36 years responded to the survey. The overall prevalence of stress, anxiety, depression, and insomnia in inpatients was 94 (7.54%), 109 (8.75%), 141 (11.32%), and 144 (11.56%), respectively. Mental distress was aggravated by COVID-19-related symptoms, comorbidities, and prolonged hospital stays. A stable internet connection was the most effective measure to reduce stress and depression. Offering inpatient with study or work facilitations, and mental health education help to ameliorate anxiety and depression. Organizing volunteering was a potential protective factor against stress.

**Conclusion:**

Humanistic care is crucial and effective for protecting against mental distress, which should be emphasized in shelter hospitals.

## 1. Introduction

The coronavirus disease 2019 (COVID-19) continues to spread around the world. According to World Health Organization COVID-19 epidemiological updates, as of 10th June 2022, over 532 million confirmed cases and over six million deaths have been reported globally. In late February of 2022, a wave of severe acute respiratory syndrome coronavirus 2 (SARS-CoV-2) clustered into the BA.2.2 sub-lineage raged unexpectedly in Shanghai, China (1). There have been 58,052 confirmed cases and 588 deaths in this wave of the pandemic in Shanghai from 26th February to 10th June 2022 according to the Shanghai Municipal Health Commission.

Based on the experiences of the COVID-19 pandemic in Wuhan in 2019, the use of shelter hospitals in quarantining infected and suspected cases of COVID-19, without symptoms or with mild symptoms, is a crucial step for public health. It is a social measure to control the spread of the disease, in addition to locking down districts with severe outbreaks, large-scale viral nucleic acid and antigen screenings, quarantining patients with close contact in hotels, and transferring special infected cases to designated hospitals. Accumulating research has established that mental distress is ubiquitous during unanticipated pandemics. This is certain to be echoed in the populations affected by the COVID-19 pandemic in Shanghai, especially inpatients quarantined in shelter hospitals (2). The potential causes of mental distress pertinent to shelter hospitals include physical distance from family, unemployment and consequent financial burden, and interference in aspects of daily life that can have significant implications for the long term such as interference in education.

In such cases, humanistic care plays a crucial role in screening for psychopathology, psychoeducation, psychosocial support, and addressing adverse psychosocial outcomes in addition to medical care. Neglected humanistic care is not only unethical but may also translate into a range of emotional reactions, unhealthy behaviors, non-compliance with shelter hospital directives, and even impairment to physical well-being among inpatients. However, evidence-based evaluations of humanistic care for the mental health of inpatients in shelter hospitals are relatively scarce. Consequently, this study conducts a retrospective, cross-sectional, observational, single-centered study validating the association of humanistic care measures with alleviating mental disorders and reducing potential risk factors among shelter hospital inpatients.

## 2. Materials and methods

### 2.1. Study design

This cross-sectional observational study conducted a hospital-based survey during the COVID-19 pandemic from 28^th^ April to 5^th^ May 2022 at the Shelter Hospital of Shanghai New International Expo Center (SNIEC) in Shanghai, China. This shelter hospital, established on 31st March 2022, consisted of 4,339 healthcare workers and hospitalized 9,519 inpatients. Accumulatively, by April 28^th^ 2022, 36,147 patients had been hospitalized here. Humanistic care measures towards inpatients were carried out by the administrative office according to the Work Program on Psychological Assistance and Social Work Services launched on 5^th^ March 2020 during the Wuhan COVID-19 pandemic. The specific humanistic care measures included active encouragement by healthcare workers, stable Internet signal offered by local telecommunication supply, study or work facilitations provided by the logistics department, online COVID-19 and mental health education offered by related specialists, and organized graffiti and emotional drawing, regular recreational activity, volunteering, and physical exercise.

The survey was conducted through online self-reported questionnaires. The primary outcome was the validation of the protective effects of humanistic care on mental distress. The secondary outcome was the occurrence rate of mental distress and potential risk factors.

This study was approved and supervised by the clinical research ethics committee of Ren Ji Hospital, School of medicine, Shanghai Jiao Tong University (No. KY2022-082-B), which is responsible for the administration of the shelter hospital. Additionally, it follows the Strengthening the Reporting of Observational studies in Epidemiology (STROBE) statement for cross-sectional studies. All participants were fully informed about this study and provided electronic informed consent prior to their enrollment. They were allowed to terminate the survey at any time and confidentiality of their information was guaranteed.

### 2.2. Participants

Participants eligible for this study included asymptomatic inpatients who had nucleic acid testing-validated SARS-CoV-2 infections and those with mild symptoms. The following patients were excluded: patients with a previous history of or diagnosed with psychological diseases by psychiatrists, with moderate or severe symptoms of COVID-19 or other unstable morbidities to be transferred out to designated hospitals, and those unable or unwilling to fill out the questionnaires. Inpatients under 18 years old were not included in this study. The family cabin in the SNIEC shelter hospital is designed for SARS-Cov-2 infected children taken care of by their parents or assigned guardians.

The sample size of patients was calculated at a 3% margin of error with a 95% confidence level.^1^ The population size of inpatients was set as 10,000. The required sample size of inpatients was calculated to be 965.

### 2.3. Data sources and measurement

Demographic, domestic, and occupational characteristics were self-reported by patients, including age, gender, marital status, children number, dependents in need of care, and hospitalization characteristics. Data on whether they were living in the family cabin was taken from the hospital management system. Participants indicated whether they were receiving a humanistic care intervention through the self-report questionnaire. This was validated by the authors.

#### 2.3.1. Questionnaires

The stress, anxiety, depression, and insomnia in inpatients were measured by the Chinese Stress Response Questionnaire-28 (SRQ-28), the Chinese version of Generalized Anxiety Disorder-7 (GAD-7), Patient Health Questionnaire-9 (PHQ-9), and Insomnia Severity Index-7 (ISI-7), respectively. All questionnaires have been granted permission for this study.

The Chinese SRQ-28 is a 28-item questionnaire whereby participants rate their responses on a 5-point Likert scale to indicate their stress response. The total score of SRQ-28 is 140, and higher scores indicate higher levels of the stress response (3). While there is no official cut-off, the moderate level of ≥84 is set as the cut-off value.

GAD-7 and its Chinese version is a 7-item questionnaire wherein participants rate their responses on a 4-point Likert scale. It is treated as a reliable and valid measure of anxiety response in the general population (2). The total score of GAD-7 is 21, and higher scores indicate a higher level of anxiety response. The cut-off of GAD-7 is set as ≥10 for moderate anxiety (4).

PHQ-9 is a 9-item questionnaire that measures depression through a 4-point Likert scale. It has a total score of 27 and the cut-off score for moderate depression is ≥10, with higher scores indicating higher levels of depression response (5). The Chinese version has been proven to be valid and reliable, and the optimal cut-off point to detect depression in Chinese outpatients is also 10 for PHQ-9 (sensitivity = 0.77, specificity = 0.76) (6).

Insomnia is assessed via the ISI, a 7-item self-report index assessing the severity of initial, middle, and late insomnia. Scores ≥15 indicate that moderate insomnia is present (7). Its Chinese version has been validated (8).

### 2.4. Statistical methods

Data analysis was performed using SPSS statistical software version 27.0.1 (IBM Corporation, Armonk, NY, United States). The significance level was set at α = 0.05, and all tests were 2-tailed. The ranked data, which were derived from the counts of each level for symptoms of stress, anxiety, depression, and insomnia are presented as numbers and percentages. Quantitative variables that were not normally distributed were presented as medians with an interquartile range (IQR). The nonparametric Mann–Whitney U test and Kruskal-Wallis test were applied to compare the severity of each symptom between two or more groups. To determine the effects of humanistic care for symptoms of stress, anxiety, depression, and insomnia, multivariable logistic regression analysis was performed, and the associations between risk factors and outcomes are presented as odds ratios (ORs) and 95% confidential intervals (CIs), after adjustment for recognized significant confounders. All respondents were required to complete the questionnaires, otherwise were excluded from the survey.

## 3. Results

In total, 1,246 out of 9,519 inpatients (response rate 13.09%) responded to the survey (Table 1). The median age with IQR of total inpatients, outside, and inside the family cabin was 36.00 [29.00, 47.00], 36.00 [28.00, 48.00], and 38.00 [33.00, 42.00], respectively. There were 565 (45.35%) females and 681 (54.65%) males in total. In the family cabin, there were more married female with children (*p* < 0.001). Additionally, inpatients in the family cabin had more dependents in need of care outside the hospital (*p* = 0.003). Education levels inside the family cabin were higher (51.95%) than outside (31.53% above K12, *p* < 0.001); however, the employment rate was comparable.

**Table 1:** Basis demographic and hospitalization characteristics of inpatients.

	Overall	Inside family cabin	Outside family cabin	
	*N* = 1,246	*N* = 231	*N* = 1,015	
Characteristics	(100%)	(18.54%)	(81.46%)	*p*
Age, year	36.00 [29.00, 47.00]	38.00 [33.00, 42.00]	36.00 [28.00, 48.00]	0.048
Gender				**<0.001**
Female	565 (45.35)	136 (58.87)	429 (42.27)	
Male	681 (54.65)	95 (41.13)	586 (57.73)	
Marital status				**<0.001**
Unmarried	368 (29.53)	16 (6.93)	352 (34.68)	
Married	878 (70.47)	215 (93.07)	663 (65.32)	
Children number				**<0.001**
0	406 (32.58)	19 (8.23)	387 (38.13)	
≥1	840 (67.42)	212 (91.77)	628 (61.87)	
Dependents in of need care				**0.030**
No	895 (71.83)	152 (65.80)	743 (73.20)	
Yes	351 (28.17)	79 (34.20)	272 (26.80)	
Education level				**<0.001**
K12 and under	806 (64.69)	111 (48.05)	695 (68.47)	
Above K12	440 (35.31)	120 (51.95)	320 (31.53)	
Employment				0.186
Employed	1,027 (82.42)	183 (79.22)	844 (83.15)	
Unemployed	219 (17.58)	48 (20.78)	171 (16.85)	
SARS-Cov-2-related symptoms				0.758
No	380 (30.50)	68 (29.44)	312 (30.74)	
Mild	866 (69.50)	163 (70.56)	703 (69.26)	
Comorbidity				0.304
No	1,077 (86.44)	205 (88.74)	872 (85.91)	
Yes	169 (13.56)	26 (11.26)	143 (14.09)	
Long-term medication				0.354
No	1,097 (88.04)	208 (90.04)	889 (87.59)	
Yes	149 (11.96)	23 (9.96)	126 (12.41)	
Traditional Chinese medicine				0.864
No	570 (45.75)	104 (45.02)	466 (45.91)	
Yes	676 (54.25)	127 (54.98)	549 (54.09)	
Nucleic acid test, times	4.00 [2.00, 7.00]	3.00 [1.00, 5.00]	5.00 [2.00, 7.00]	**<0.001**
Hospital length of stay, day	6.00 [3.00, 9.00]	5.00 [3.00, 7.00]	6.00 [4.00, 9.00]	**<0.001**

Bold values mean the *p*-value is significant.

Inpatients in both groups presented similar rates of SARS-Cov-2-related symptoms, comorbidities, and long-term medication and received traditional Chinese medicine without significant differences. Inpatients were administered SARS-Cov-2 nucleic acid test on the third day after hospitalization. The median nucleic acid test times and hospital length of stay with IQR was 4.00 [2.00, 7.00] and 6.00 [3.00, 9.00] days overall, 3.00 [1.00, 5.00] and 5.00 [3.00, 7.00] days in the family cabin, and 5.00 [2.00, 7.00] and 6.00 [4.00, 9.00] days outside the family cabin, respectively (*p* < 0.001).

The overall prevalence of stress, anxiety, depression, and insomnia of inpatients was 94 (7.54%), 109 (8.75%), 141 (11.32%), and 144 (11.56%), respectively (Table 2). In our sample, females were prone to anxiety (57.80% vs. 44.15%, *p* = 0.008) and depression (55.32% vs. 44.07%, *p* = 0.015) in comparison with males. Inpatients with children were reportedly more resistant to insomnia (59.72% vs. 68.42%, *p* = 0.045). Additionally, having more dependents in need of care, a higher level of education and longer hospital stays were strongly associated with an increased risk of anxiety, depression, and insomnia. Unemployment was also a potential risk factor for depression (*p* = 0.022), while SARS-COV-2-related symptoms were strongly related to depression and insomnia (both *p* < 0.001). Inpatients with comorbidities were more likely to experience anxiety (*p* = 0.024) and insomnia (*p* = 0.005). The comorbidities included diabetes, hypertension, cardiovascular disease, and other specific chronic diseases.

**Table 2:** The prevalence of mental disorders among inpatients.

	SRQ			GAD			PHQ			ISI		
	Normal mild	Moderate severe	*p*	Normal mild	Moderate severe	*p*	Norma mild	Moderate severe	*p*	Normal mild	Moderate severe	*p*
Characteristics	*N* = 1,152 (92.46%)	*N* = 94 (7.54%)		*N* = 1,137 (91.25%)	*N* = 109 (8.75%)		*N* = 1,105 (88.68%)	*N* = 141 (11.32%)		*N* = 1,102 (88.44%)	*N* = 144 (11.56%)	
Age, year	36.00 [29.00, 47.00]	35.00 [28.00, 44.00]	0.546	36.00 [29.00, 47.00]	36.00 [30.00, 41.00]	0.593	36.00 [29.00, 47.00]	37.00 [29.00, 43.00]	0.462	36.00 [29.00, 47.00]	35.00 [28.00, 41.25]	0.124
Gender												
Female	516 (45.70)	49 (41.88)	0.488	502 (44.15)	63 (57.80)	**0.008**	487 (44.07)	78 (55.32)	**0.015**	492 (44.65)	73 (50.69)	0.200
Male	613 (54.30)	68 (58.12)		635 (55.85)	46 (42.20)		618 (55.93)	63 (44.68)		610 (55.35)	71 (49.31)	
Marital status												
Unmarried	335 (29.67)	33 (28.21)	0.822	341 (29.99)	27 (24.77)	0.302	328 (29.68)	40 (28.37)	0.823	318 (28.86)	50 (34.72)	0.176
Married	794 (70.33)	84 (71.79)		796 (70.01)	82 (75.23)		777 (70.32)	101 (71.63)		784 (71.14)	94 (65.28)	
Children number												
0	368 (32.60)	38 (32.48)	1.000	375 (32.98)	31 (28.44)	0.390	358 (32.40)	48 (34.04)	0.767	348 (31.58)	58 (40.28)	**0.045**
≥1	761 (67.40)	79 (67.52)		762 (67.02)	78 (71.56)		747 (67.60)	93 (65.96)		754 (68.42)	86 (59.72)	
Dependents in need of care												
No	819 (72.54)	76 (64.96)	0.103	848 (74.58)	47 (43.12)	**<0.001**	829 (75.02)	66 (46.81)	**<0.001**	823 (74.68)	72 (50.00)	**<0.001**
Yes	310 (27.46)	41 (35.04)		289 (25.42)	62 (56.88)		276 (24.98)	75 (53.19)		279 (25.32)	72 (50.00)	
Education level												
K12 and under	732 (64.84)	74 (63.25)	0.810	756 (66.49)	50 (45.87)	**<0.001**	737 (66.70)	69 (48.94)	**<0.001**	738 (66.97)	68 (47.22)	**<0.001**
Above K12	397 (35.16)	43 (36.75)		381 (33.51)	59 (54.13)		368 (33.30)	72 (51.06)		364 (33.03)	76 (52.78)	
Employment												
Employed	935 (82.82)	92 (78.63)	0.315	945 (83.11)	82 (75.23)	0.053	921 (83.35)	106 (75.18)	**0.022**	915 (83.03)	112 (77.78)	0.150
Unemployed	194 (17.18)	25 (21.37)		192 (16.89)	27 (24.77)		184 (16.65)	35 (24.82)		187 (16.97)	32 (22.22)	
Family cabin												
No	917 (81.22)	98 (83.76)	0.584	930 (81.79)	85 (77.98)	0.396	903 (81.72)	112 (79.43)	0.587	896 (81.31)	119 (82.64)	0.785
Yes	212 (18.78)	19 (16.24)		207 (18.21)	24 (22.02)		202 (18.28)	29 (20.57)		206 (18.69)	25 (17.36)	
SARS-COV-2-related symptoms												
No	344 (30.47)	36 (30.77)	1.000	355 (31.22)	25 (22.94)	0.092	360 (32.58)	20 (14.18)	**<0.001**	356 (32.30)	24 (16.67)	**<0.001**
Mild	785 (69.53)	81 (69.23)		782 (68.78)	84 (77.06)		745 (67.42)	121 (85.82)		746 (67.70)	120 (83.33)	
Comorbidity												
No	976 (86.45)	101 (86.32)	1.000	991 (87.16)	86 (78.90)	**0.024**	963 (87.15)	114 (80.85)	0.054	964 (87.48)	113 (78.47)	**0.005**
Yes	153 (13.55)	16 (13.68)		146 (12.84)	23 (21.10)		142 (12.85)	27 (19.15)		138 (12.52)	31 (21.53)	
Long-term medication												
No	997 (88.31)	100 (85.47)	0.453	1,006 (88.48)	91 (83.49)	0.168	978 (88.51)	119 (84.40)	0.201	976 (88.57)	121 (84.03)	0.149
Yes	132 (11.69)	17 (14.53)		131 (11.52)	18 (16.51)		127 (11.49)	22 (15.60)		126 (11.43)	23 (15.97)	
Traditional Chinese Medicine												
No	514 (45.53)	56 (47.86)	0.700	516 (45.38)	54 (49.54)	0.464	508 (45.97)	62 (43.97)	0.719	506 (45.92)	64 (44.44)	0.807
Yes	615 (54.47)	61 (52.14)		621 (54.62)	55 (50.46)		597 (54.03)	79 (56.03)		596 (54.08)	80 (55.56)	
Nucleic acid test times, times	4.00 [2.00, 7.00]	4.00 [2.00, 7.00]	0.929	4.00 [2.00, 7.00]	5.00 [2.00, 7.00]	0.376	4.00 [2.00, 7.00]	5.00 [3.00, 7.00]	0.258	4.00 [2.00, 7.00]	5.00 [2.00, 7.00]	0.237
Hospital length of stay, day	6.00 [3.00, 9.00]	6.00 [3.00, 9.00]	0.558	6.00 [3.00, 9.00]	7.00 [4.00, 10.00]	**0.019**	6.00 [3.00, 9.00]	7.00 [4.00, 10.00]	**0.004**	6.00 [3.00, 9.00]	7.00 [4.00, 10.00]	**0.030**

Bold values mean the *p*-value is significant.

Among humanistic care towards inpatients, a stable internet connection appeared to be the most effective means to reduce stress [OR = 0.385, CI (0.235, 0.631), *p* < 0.001], anxiety [OR = 0.491, (0.294, 0.818), *p* = 0.006], and depression [OR = 0.575, CI (0.350, 0.944), *p* = 0.029], but not insomnia (Table 3). Offering inpatients study or work facilities and mental health education helped to ameliorate anxiety [OR = 0.637, CI (0.414, 0.980), *p* = 0.040 and OR = 0.598, CI (0.360, 0.995), *p* = 0.048] and depression [OR = 0.478, CI (0.322, 0.710), *p* < 0.001 and OR = 0.546, CI (0.344, 0.866), *p* = 0.010]. Organizing volunteering activities by cabin administrators regularly was a potential protective factor against stress [OR = 0.578, CI (0.346, 0.968), *p* = 0.037].

**Table 3:** Humanistic care effects on mental disorders among inpatients.

	SRQ				GAD				PHQ				ISI			
	OR	CI		*p*	OR	CI		*p*	OR	CI		*p*	OR	CI		*p*
Active encouragement																
No	1 [Reference]				1 [Reference]				1 [Reference]				1 [Reference]			
Yes	1.170	0.725	1.888	0.520	0.789	0.491	1.268	0.328	0.705	0.450	1.104	0.126	0.827	0.531	1.288	0.401
Graffiti and emotional drawing																
No	1 [Reference]				1 [Reference]				1 [Reference]				1 [Reference]			
Yes	0.867	0.542	1.388	0.552	1.171	0.712	1.927	0.534	1.321	0.816	2.138	0.257	1.177	0.742	1.866	0.489
Stable Internet signal																
No	1[Reference]				1[Reference]				1[Reference]				1[Reference]			
Yes	0.385	0.235	0.631	**<0.001**	0.491	0.294	0.818	**0.006**	0.575	0.350	0.944	**0.029**	0.641	0.393	1.046	0.075
Study or work facilitations																
No	1 [Reference]				1 [Reference]				1 [Reference]				1 [Reference]			
Yes	0.996	0.659	1.506	0.986	0.637	0.414	0.980	**0.040**	0.478	0.322	0.710	**<0.001**	0.828	0.563	1.217	0.336
COVID-19 education																
No	1 [Reference]				1 [Reference]				1 [Reference]				1 [Reference]			
Yes	0.980	0.536	1.792	0.947	0.828	0.464	1.477	0.522	1.735	0.957	3.144	0.070	0.935	0.534	1.638	0.816
Mental health education																
No	1 [Reference]				1 [Reference]				1 [Reference]				1 [Reference]			
Yes	0.901	0.555	1.464	0.674	0.598	0.360	0.995	**0.048**	0.546	0.344	0.866	**0.010**	0.807	0.517	1.257	0.342
Regular recreational activity																
No	1 [Reference]				1 [Reference]				1 [Reference]				1 [Reference]			
Yes	0.732	0.439	1.221	0.232	0.912	0.530	1.569	0.740	1.137	0.693	1.865	0.612	0.788	0.481	1.290	0.344
Organizing volunteering																
No	1 [Reference]				1 [Reference]				1 [Reference]				1 [Reference]			
Yes	0.578	0.346	0.968	**0.037**	1.043	0.639	1.701	0.868	0.895	0.566	1.414	0.634	0.952	0.610	1.484	0.827
Organizing physical exercise																
No	1 [Reference]				1 [Reference]				1 [Reference]				1 [Reference]			
Yes	0.866	0.553	1.356	0.529	0.844	0.531	1.340	0.471	0.892	0.585	1.361	0.596	0.878	0.583	1.323	0.535

Patient’s characteristics with *p* < 0.05 in the univariable analysis (Table 2) were adjusted in respective logistic regression models. SRQ had no statistically significant factors in univariate analysis. GAD was adjusted for gender, dependents in need of care, education level, comorbidity and hospital length of stay. PHQ was adjusted for gender, dependents in need of care, education level, employment, SARS-COV-2-related symptoms and hospital length of stay. ISI was adjusted for children number, dependents in need of care, education level, SARS-COV-2-related symptoms, comorbidity and hospital length of stay.

Bold values mean the *p*-value is significant.

## 4. Discussion

To avoid quarantining children alone, results indicate that being placed in a family cabin is by all means very necessary in the SNIEC shelter hospital. In addition, hospitalization in the family cabin was found to be significantly related to shorter hospital length of stay, although it is not associated with relief of mental disorders and SARS-COV-2 symptoms. The family cabin is both beneficial to inpatients and their family members. The physical presence of the caregiver in the rehabilitation setting of family glass cabin was reported to not only increases acute brain injury patients’ functional recovery, but also reduces caregivers’ anxiety and emotional burden (9).

**Figure 1:**
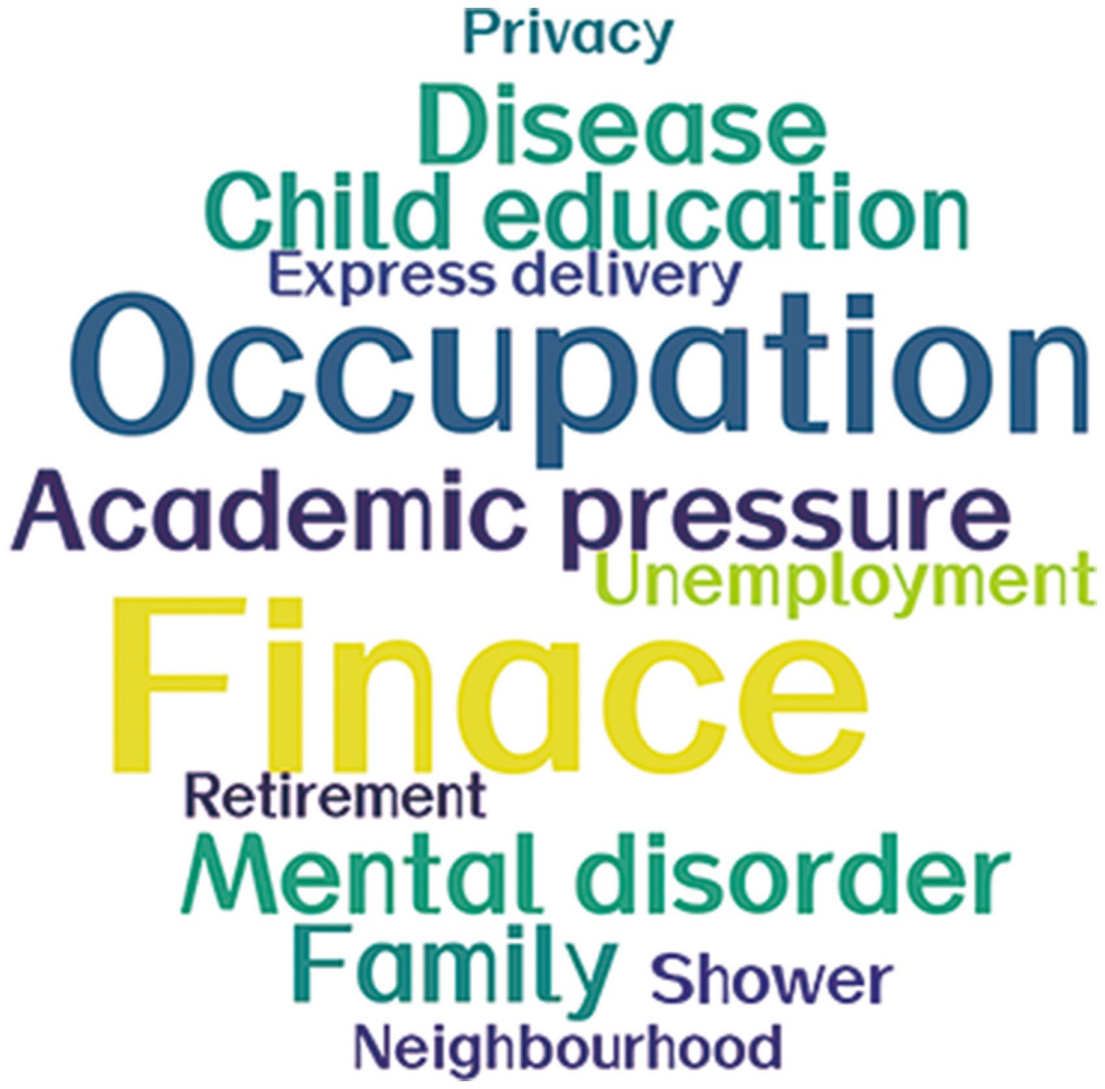
Word cloud of the major stressors among inpatients. Major stressors are presented as a word cloud. Font size is positively correlated with proportion.

Major reported stressors of inpatients included concerns about finances, occupation, children’s education, family issues (Figure 1). These findings are in line with existing research. For instance, Pierce et al. identified predictive factors for deteriorating mental health, which included infection with SARS-CoV-2, local lockdown, and financial difficulties. They compared their findings with pre-pandemic data from the United Kingdom’s adult population (10). Inpatients with SARS-COV-2-related symptoms, comorbidities, and prolonged hospital length of stay are more susceptible to mental disorders. Obesity and a wide range of comorbidities are risk factors for long COVID (11). These inpatients warrant special attention in treatments and nursing care that is tailored to their symptoms and comorbidities. Inpatients with moderate symptoms or above, and unstable comorbidities are screened before hospitalization, diagnosed during hospitalization, and transferred out of the shelter hospital to designated hospitals with sophisticated medical equipment. Moreover, dependents in need of care outside the shelter hospital are at strong risk of mental distress. Collaborations between the government, the shelter hospital, and community social workers have been established to ensure they are not unattended; particularly, women and those with higher levels of education and unemployment are screened as they have higher risks of mental distress.

We also found that inpatients with children were less vulnerable to insomnia. This may be explained by previous experiences of interrupted sleep at night when looking after children. However, a national longitudinal cohort study in the United Kingdom elaborated that greater increases in mental disorders are observed in young adults, especially women (< 35 years), and those living with preschool children (< 5 years) (12). Additionally, having a stable internet connection was a strong protective factor for reducing stress, anxiety, and depression. This facilitates online research based on patients’ generated data, as well as popularizing knowledge of COVID-19 (13). Mental health education was established by watching the relevant online recorded video. These were demonstrated to be linked with reduced anxiety and depression. A similar effect was noted in offering study or work facilities, such as power supply, desks, and pens. Volunteering activities were also organized by cabin administrators to help healthcare workers in goods transportation and distribution, order maintenance, garbage clearance, and other logistical roles—these were determined to relieve anxiety. Similarly, Chan et al. also demonstrated that volunteering among Chinese older adults during COVID-19 was related to fewer depressive and anxiety symptoms and higher self-esteem (14).

### 4.1. Limitations

This study has several limitations. Firstly, this single-center study has a limited sample size of 1,246 inpatients, which limits the generalizability of the findings to other regions. Secondly, humanistic care was defined and measured as a binary variable of whether care was received or not. In-depth quantification and standardization of measuring humanistic care are warranted for future studies. Thirdly, the observational period of this cross-sectional observational study was 8 days, which was relatively short; the long-term manifestation of inpatients’ mental disorders is worth further investigation.

## 5. Conclusion

Symptoms of mental disorders were prevalent among inpatients in the shelter hospital. They are aggravated by COVID-19-related symptoms, comorbidities, and prolonged hospital stays. After screening and adjusting for potential risk factors, humanistic care is crucial in ameliorating mental distress.

## Data availability statement

The raw data supporting the conclusions of this article will be made available by the authors, without undue reservation.

## Ethics statement

The studies involving human participants were reviewed and approved by Shanghai Jiaotong University School of Medicine, Renji Hospital Ethics Committee. The patients/participants provided their written informed consent to participate in this study. Written informed consent was obtained from the individual(s) for the publication of any potentially identifiable images or data included in this article.

## Author contributions

DX and MC conducted the interview. JQ, YL, and JZ designed the study as supervisors. HH and JY recorded the interview data for JW to analyze. WZ and MJC translated and designed the questionnaires. WF, YZ, and ZF completed the application to ethics committee. YS, ZY, and CS wrote this manuscript. All authors contributed to the article and approved the submitted version.

## Funding

This work was supported by Shanghai Health Science Popularization Special Program (JKKPZX-2022-A15); and Hospital-pharma Integration Project on Innovation-boosting Expertise Training (SHDC2022CRS030).

## Conflict of interest

The authors declare that the research was conducted in the absence of any commercial or financial relationships that could be construed as a potential conflict of interest.

The reviewer LH declared a shared affiliation with the authors to the handling editor at the time of review.

## Publisher’s note

All claims expressed in this article are solely those of the authors and do not necessarily represent those of their affiliated organizations, or those of the publisher, the editors and the reviewers. Any product that may be evaluated in this article, or claim that may be made by its manufacturer, is not guaranteed or endorsed by the publisher.

## Footnotes

^1^ https://www.qualtrics.com/blog/calculating-sample-size/

## References

[1] Zhang X Zhang W Chen S. Shanghai's life-saving efforts against the current omicron wave of the Covid-19 pandemic. Lancet. (2022) 399:2011–2. doi: 10.1016/S0140-6736(22)00838-8, PMID: 35533708 PMC9075855

[2] Wang Y Zhang M Yin Q Wang Y Yang P Hu C. Psychological responses of the patients in cabin hospital to the Covid-19 outbreak: a comparative epidemiologic analysis. Front Psychol. (2021) 12:641167. doi: 10.3389/fpsyg.2021.641167, PMID: 34322052 PMC8312570

[3] Wang Y Wang P. Perceived stress and psychological distress among Chinese physicians: the mediating role of coping style. Medicine (Baltimore). (2019) 98:e15950. doi: 10.1097/MD.0000000000015950, PMID: 31169719 PMC6571215

[4] Lowe B Decker O Muller S Brahler E Schellberg D Herzog W. Validation and standardization of the generalized anxiety disorder screener (gad-7) in the general population. Med Care. (2008) 46:266–74. Epub 2008/04/05. doi: 10.1097/MLR.0b013e318160d093, PMID: 18388841

[5] Manea L Gilbody S McMillan D. A diagnostic Meta-analysis of the patient health Questionnaire-9 (Phq-9) algorithm scoring method as a screen for depression. Gen Hosp Psychiatry. (2015) 37:67–75. doi: 10.1016/j.genhosppsych.2014.09.009 25439733

[6] Xiong N Fritzsche K Wei J Hong X Leonhart R Zhao X. Validation of patient health questionnaire (Phq) for major depression in Chinese outpatients with multiple somatic symptoms: a multicenter cross-sectional study. J Affect Disord. (2015) 174:636–43. doi: 10.1016/j.jad.2014.12.042, PMID: 25576931

[7] Morin CM Belleville G Belanger L Ivers H. The insomnia severity index: psychometric indicators to detect insomnia cases and evaluate treatment response. Sleep. (2011) 34:601–8. Epub 2011/05/03. doi: 10.1093/sleep/34.5.601, PMID: 21532953 PMC3079939

[8] Zhang B Zhang L Chen S Luo X Dhirendra P Lin Q. The effect of E-aid cognitive behavioral therapy in treating chronic insomnia disorder: an open-label randomized controlled trial. Chin J Psychiatry. (2019) 52:373–8. doi: 10.3760/cma.j.issn.1006-7884.2019.06.003

[9] De Luca R Rifici C Pollicino P Parisi S Bonanno M Torregrossa W. Is the "family glass cabin" useful to safely allow inpatient-caregiver interaction in the Covid-19 era? A pilot study on severe acquired brain injury. J Clin Med. (2022) 11:1623. doi: 10.3390/jcm11061623, PMID: 35329947 PMC8950736

[10] Pierce M McManus S Hope H Hotopf M Ford T Hatch SL. Mental health responses to the Covid-19 pandemic: a latent class trajectory analysis using longitudinal Uk data. Lancet Psychiatry. (2021) 8:610–9. doi: 10.1016/S2215-0366(21)00151-6, PMID: 33965057 PMC9764381

[11] Subramanian A Nirantharakumar K Hughes S Myles P Williams T Gokhale KM. Symptoms and risk factors for long Covid in non-hospitalized adults. Nat Med. (2022) 28:1706–14. doi: 10.1038/s41591-022-01909-w, PMID: 35879616 PMC9388369

[12] Pierce M Hope H Ford T Hatch S Hotopf M John A. Mental health before and during the Covid-19 pandemic: a longitudinal probability sample survey of the Uk population. Lancet Psychiatry. (2020) 7:883–92. doi: 10.1016/s2215-0366(20)30308-4, PMID: 32707037 PMC7373389

[13] Reuter K Deodhar A Makri S Zimmer M Berenbaum F Nikiphorou E. The impact of the Covid-19 pandemic on people with rheumatic and musculoskeletal diseases: insights from patient-generated data on social media. Rheumatology (Oxford). (2021) 60:SI77. doi: 10.1093/rheumatology/keab174, PMID: 33629107 PMC7928589

[14] Chan W Chui CHK Cheung JCS Lum TYS Lu S. Associations between volunteering and mental health during Covid-19 among Chinese older adults. J Gerontol Soc Work. (2021) 64:599–612. doi: 10.1080/01634372.2021.1904079, PMID: 33769224

